# Macrocycle-Based Supramolecular Drug Delivery Systems: A Concise Review

**DOI:** 10.3390/molecules29163828

**Published:** 2024-08-12

**Authors:** Yanrui Yang, Pengcheng Li, Haibo Feng, Rui Zeng, Shanshan Li, Qixiong Zhang

**Affiliations:** 1 College of Pharmacy, Key Laboratory of Research and Application of Ethnic Medicine Processing and Preparation on the Qinghai Tibet Plateau, Southwest Minzu University, Chengdu 610041, China; 2 College of Animal Husbandry and Veterinary Medicine, Southwest Minzu University, Chengdu 610041, China; 3 Department of Pharmacy, Personalized Drug Therapy Key Laboratory of Sichuan Province, Sichuan Academy of Medical Science & Sichuan Provincial People’s Hospital, School of Medicine, University of Electronic Science and Technology of China, Chengdu 610072, China; 4 Department of Pharmacy, Sichuan Provincial People’s Hospital Chuandong Hospital & Dazhou First People’s Hospital, Dazhou 635000, China

**Keywords:** macrocyclic compounds, host–guest interaction, supramolecular chemistry, drug delivery systems, bioavailability

## Abstract

Efficient delivery of therapeutic agents to the lesion site or specific cells is an important way to achieve “toxicity reduction and efficacy enhancement”. Macrocycles have always provided many novel ideas for drug or gene loading and delivery processes. Specifically, macrocycles represented by crown ethers, cyclodextrins, cucurbit[n]urils, calix[n]arenes, and pillar[n]arenes have unique properties, which are different cavity structures, good biocompatibility, and good stability. Benefited from these diverse properties, a variety of supramolecular drug delivery systems can be designed and constructed to effectively improve the physical and chemical properties of guest molecules as needed. This review provides an outlook on the current application status and main limitations of macrocycles in supramolecular drug delivery systems.

## 1. Introduction

Drugs with low water solubility often fail to achieve ideal diagnostic and therapeutic effects due to their low bioavailability and non-specific distribution. Compared with traditional drug delivery strategies, novel drug delivery systems (NDDSs) based on different materials, such as host–guest inclusion complexes [1], polymer micelles [2], polymeric nanoparticles [3], inorganic nanoparticles [4], hydrogels [5], and liposomes [6], can effectively improve the loading efficiency, avoid drug degradation, and reduce toxicity and side effects. In addition, active and passive targeting to specific sites can be achieved by introducing targeting units [7,8] and stimuli-responsive groups [9,10], respectively. Therefore, the development of intelligent drug delivery systems (DDSs) with excellent performance has attracted extensive attention both in the fields of chemistry and pharmacy.

**Figure 1:**
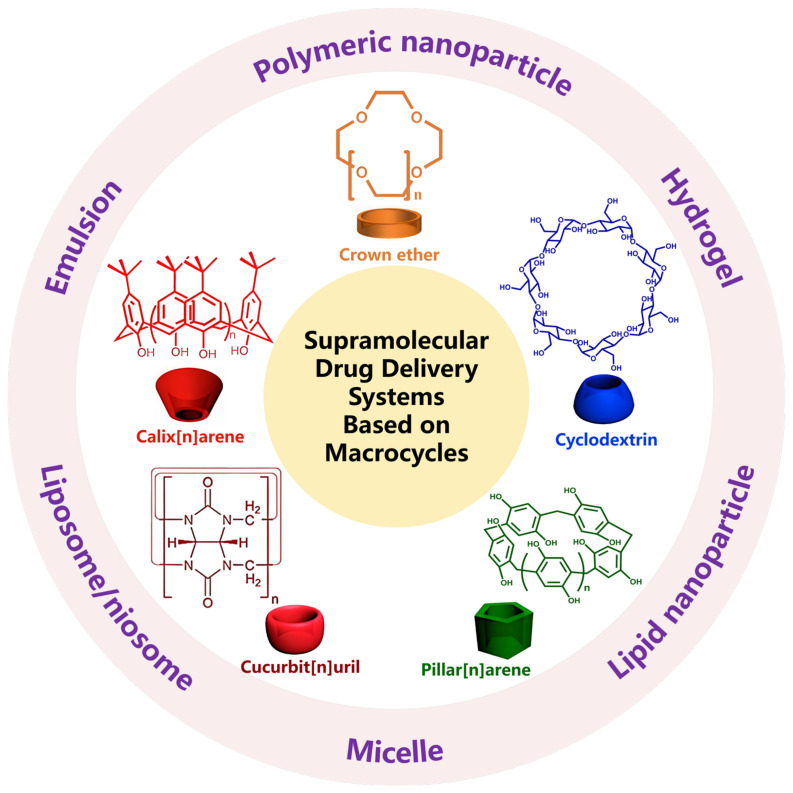
SDDSs based on various macrocycles.

It is worth noting that as the study develops in depth, the research scope of macrocycles continues to expand, from the initial crown ethers (CEs) and cyclodextrins (CDs) to cucurbit[n]urils (CB[n]s), calix[n]arenes (C[n]As), and pillar[n]arenes (P[n]As) [11]. Accordingly, the research of supramolecular drug delivery systems (SDDSs) based on macrocycles is constantly being updated. Specifically, the cavities of macrocycles could be effectively complexed with different types of guest molecules, thereby constructing varieties of supramolecular assemblies [12,13,14]. SDDSs constructed based on these dynamic host–guest interactions can achieve reversible changes in structure, morphology, and functions under external stimulations [14]. That is beneficial for the targeted and controlled release of payloads, thus reducing damage to normal tissues/cells and enhancing diagnostic and therapeutic effects. Additionally, using macrocycles as host molecules could improve the bioavailability of payloads by significantly enhancing their water solubility and loading capacity. In this review, we will introduce the current status of SDDSs based on five kinds of macrocycles, hoping to provide new enlightenments for their applications in the biomedical field (Figure 1).

## 2. Crown Ether-Based DDSs

**Figure 2:**
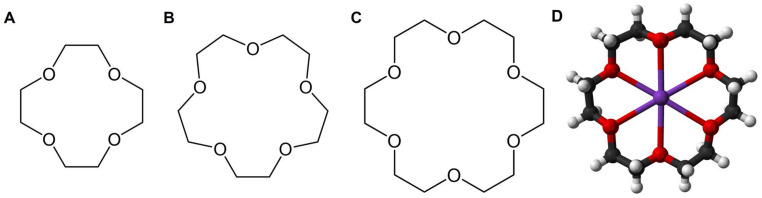
A variety of CEs. (**A**), 12-crown-4 (12C4). (**B**), 15-crown-5 (15C5). (**C**), 18-crown-6 (18C6). (**D**), The binding model of 18C6 and K^+^.

Crown ether (CE) was firstly synthesized by Peterson in 1967 [15]. Since then, CEs have made great progress in analytical chemistry, polymer chemistry, organic chemistry, and medicinal chemistry due to their unique ring structures and complexation to metal ions. Specifically, the oxygen atoms in the CE structure determined its ability to complex a wide range of cations (Figure 2) [14,16,17]. Although CE compounds are simple in structure, their properties are surprisingly similar to those of natural ionophores. It is worth mentioning that the oxygen atoms in CEs can also be replaced by other atoms, such as nitrogen atoms, which also exhibit coordination capabilities [18]. Due to the special structure of CEs, the complexes formed between CEs and ions are amphiphilic, with ions forming hydrophilic centers and polyether structures forming hydrophobic exteriors [19]. The amphiphilicity of complexes made CEs achieve significant progress in SDDSs [20]. Although CEs are easily modifiable, the main problems that hinder the applications of CEs in the field of biomedicine are their relatively high price and certain toxicity.

### 2.1. Ion Transporters or Ion Containers Based on CEs

Ion transport is an important part of the life processes. Some rare diseases such as cystic fibrosis [21], Bart syndrome [22], Dent’s Disease [23], or the common heart ion channel disease [24] are caused by ion transport dysfunction. For such diseases, either an ion or its effective transport can be considered to have a specific therapeutic effect [25,26]. Therefore, the development of such ion transport systems has potential application prospects for the treatment of ion channel diseases [25]. As mentioned above, the most prominent characteristic of CEs is the good binding and transporting capacity of cations, so a variety of different CE-based ion transporters can be constructed.

In order to simulate the process of life, CEs are usually conjugated with other functional groups to form amphiphilic compounds, which can be embedded into the membrane structure of liposomes or vesicles to act as ion transporters. For example, dimeric conjugates Azo-2C5 and Azo-2C6 were prepared by reacting benzo-15-crown-5 (B15C5) or benzo-18-crown-6 (B18C6) with thiourea compounds with lipophilic azobenzene as a linker, respectively [27]. Azo-2C5 and Azo-2C6 were embedded in the membrane of large unilamellar vesicles (LUVs) composed of palmitoyl oleyl phosphatidyl choline (POPC), and the transport effects of Na^+^, K^+^, and Cl^−^ were detected by loading pH-sensitive fluorescent probes (HPTS) in the LUV. The authors demonstrate that both Azo-2C5 and Azo-2C6 can transport Cl^−^ across membranes with considerable activity, and that the cavity of Azo-2C5 can accommodate Na^+^ well, thus achieving synchronous transport of Na^+^ and Cl^−^. It is worth noting that when there is a high concentration of environmental K^+^, Azo-2C5 and Azo-2C6 are mainly distributed outside the membrane, thus inhibiting their transport activity. On the contrary, Azo-2C5 and Azo-2C6 can better promote the efflux of K^+^ that only exists inside the LUV.

**Figure 3:**
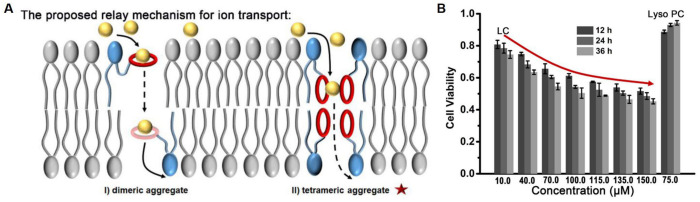
The synthetic phospholipid derivative (LC) based on B18C6 can realize cationic transport (**A**) and inhibit the viability of HeLa cells (**B**) [28]. Reprinted with permission from *Front. Chem.* **2021**, *9*, 667472. Copyright 2021, Frontiers.

Another study was inspired by the structure of natural phospholipids on cell membranes to prepare an artificially synthesized benzo-18-crown-6 (B18C6) modified phospholipid derivative (LC), which can work together with natural phospholipids to form a liposome containing ion transport channels [28]. Similarly, using HPTS as a probe, it was demonstrated that this photosensitive derivative containing B18C6 has excellent transport activity, with an EC_50_ value of 11.2 μM for K^+^. By combining channel current signals, it was revealed that LC can form a dimeric or tetrameric ion transporter through relay transport (Figure 3A). More interestingly, the LC showed some cytotoxicity to Hela cells (Figure 3B). This suggests that CE-based ion transporters can not only treat diseases caused by defective ion transport, but also have biological effects on tumor cells or other cells by influencing this process.

In addition to the construction of ion transporters, CEs can also serve as ion carriers. For example, CEs can directly carry certain atoms with therapeutic effects (such as radioactive isotopes). Actinium-225 (^225^Ac), which can produce radioactive alpha particles, can be chelated by an 18-member heterocyclic CE (called Macropa) [18]. Excitingly, compared to the traditional chelating agent DOTA (which requires a reaction temperature of 60–80 °C), the Macropa can chelate ^225^Ac at room temperature and has significant advantages in terms of synthesis simplicity. Furthermore, coupling human monoclonal antibody GC33 (ligand of GPC3) onto Macropa (named ^225^Ac-Macropa-GC33) can achieve targeted radiotherapy of GPC3-overexpressing HepG2 cancer cells.

CEs can not only directly serve as containers for therapeutic drugs, but also assist in improving the efficacy of certain drug treatments related to ion concentration. For example, researchers have found that the permeability and delivery efficiency of the drug to the eye tissue are largely dependent on the tight junctions in the corneal epithelium. The concentration of Ca^2+^ is positively correlated with this tight junction [29]. Based on this, CE is able to capture Ca^2+^ through chelation, thereby relaxing these tight connections and ultimately promoting increased drug penetration into the cornea [30]. The abilities of 12-crown-4 (12C4), 15-crown-5 (15C5), and 18-crown-6 (18C6) to capture Ca^2+^ were compared in isolated bovine corneas, and it was concluded that CE with smaller cavity size showed a better binding effect. Further, with the help of the host–guest interaction, 12C4 was able to maximize riboflavin penetration. Therefore, 12C4 has the potential to become an excellent penetrant enhancer to improve the bioavailability and efficacy of drugs for eye diseases [30]. Another report presented similar results: a nanovesicle based on Span-60 and 18C6, namely Crownsomes, was prepared [31]. And phenytoin sodium, a re-epithelialization promoting drug, was loaded. Research has confirmed that the use of CE to recognize and capture Ca^2+^ in the cornea improves the permeability of ex vivo corneas to phenytoin sodium (1.78 times higher than drug suspension), and also achieves better therapeutic effects in the alkaline induced corneal injury rabbit model [31].

The above results suggest that CEs can not only act as ion transporters or containers to achieve the adsorption or concentration regulation of cations, but also synergize with other drugs to improve therapeutic efficacy.

### 2.2. CE-Based NDDSs

It is well known that micro–nano-scale drug carriers have significant advantages in improving drug bioavailability and targeting ability. The container properties of CEs for cations enable it to achieve a wide variety of NDDSs.

#### 2.2.1. Improve the Encapsulation Efficiency of Nucleic Acid Drugs

Due to the negative charge of nucleic acids, the drug encapsulation efficiency often depends on the cation abundance of the carrier material. It has been reported that two different CE-modified neutral lipid compounds (12C4L and 15C5L) were prepared, and corresponding hybrid liposomes were constructed with natural phospholipid POPC, respectively [32]. On the one hand, the lipid tails of 12C4L and 15C5L can be inserted into the POPC bilayer, promoting the destabilization of Triton X-100 at high surfactant concentrations. More importantly, these two hybrid liposomes can actively chelate Ca^2+^, leading to increased cation concentration. Thereby, the DNA encapsulation efficiency was improved. There are two significant advantages of those hybrid liposomes. One is that neutral lipids can be used to avoid the hemolytic toxicity of cationic lipids. The other is that the drug loading and encapsulation efficiency of nucleic acid drugs are controllable by adjusting the concentration of Ca^2+^ [32].

#### 2.2.2. Construct Responsive DDSs

Responsive carrier materials have been widely recognized and applied in the design of DDSs. Through the chelation of cations, a variety of CE-based responsive drug delivery systems can also be constructed. For instance, CE can act as a “gatekeeper” to achieve a responsive “ON/OFF” effect. A series of Fe_3_O_4_@SiO_2_@meso-SiO_2_@CEs based on different CE molecules were prepared to load a hydrophobic drug (DOX) [33]. Initially, DOX was maintained in mesoporous silicon, while the outermost hydrophilic part CEs prevented its release by chelating with exogenous cations (Na^+^, Cs^+^). Under ultrasound or in an acidic environment (pH 4.0), the interactions between CEs and cations were disrupted and resulted in controlled release of DOX [33].

**Figure 4:**
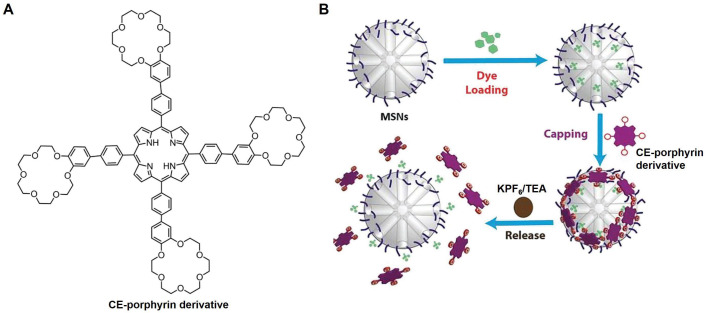
CE acts as a gatekeeper to achieve responsive drug release. (**A**), The chemical structure of 18C6-modified porphyrin derivatives. (**B**), The CE–porphyrin derivative acts as a gatekeeper for MSN to achieve KPF_6_- or TEA-responsive drug release [34]. Reprinted with permission from *J. Porphyr. Phthalocyanines* **2021**, *25*, 95–101. Copyright 2021, World Scientific Publishing Co. Pte Ltd.

In another study, as shown in Figure 4, a tetrameric porphyrin derivative modified with four 18C6 moieties was reported and used as a gatekeeper to prepare a mesoporous silica nanoparticle (MSN) encapsulated with fluorescein dye [34]. This CE–porphyrin derivative firstly chelated with ammonium cations (–NH_3_^+^) on the outer surface of MSN and acted as a gatekeeper. Under the action of potassium hexafluorophosphate (KPF_6_), K^+^ with higher affinity for CE competitively replaced ammonium cations. That makes the fixed CE porphyrin derivatives on the outer surface of MSN be free, thereby achieving the release of fluorescein dye from the pores. Naturally, the deprotonation of –NH_3_^+^ by adding triethylamine also allowed the CE–porphyrin derivatives to detach from the outer surface of MSN and eventually triggered drug release. Therefore, it can be seen that a versatile responsive gatekeeper (ultrasonic, pH, other competitive ligands, etc.) can be built based on CEs to achieve controlled drug release.

In addition to acting as a gatekeeper, CEs can also be involved as grafts to build polymer-based DDSs. Specifically, K^+^-responsive CE-mediated nano-scale DDS can be prepared. For example, a B18C6-grafted amphiphilic copolymer (PLENB) has been synthesized [35]. By solvent evaporation, PLENB can self-assemble into micelles and hydrophobic DOX could be loaded in the hydrophobic core. With the chelation of K^+^ (150 mM) and CE, the hydrophilicity of PLENB increased significantly, resulting in swelling of PLENB micelles and ultimately inducing the release of DOX [35]. However, it is worth mentioning that for water-soluble drugs, the relationship between the presence of cations and drug release tendency is opposite. As previously reported, poly(*N*-isopropylacrylamide-co-benzo-18-crown [6]-acrylamide) can be self-assembled into molecular-recognizing microcapsules with CE as the pore. It is precisely because of the presence of Ba^2+^ that the pore is closed by the chelation of Ba^2+^ with CE that makes the release rate of loaded vitamin B12 be greatly slowed down [36].

#### 2.2.3. Perfluoro-CE-Based DDSs

**Figure 5:**
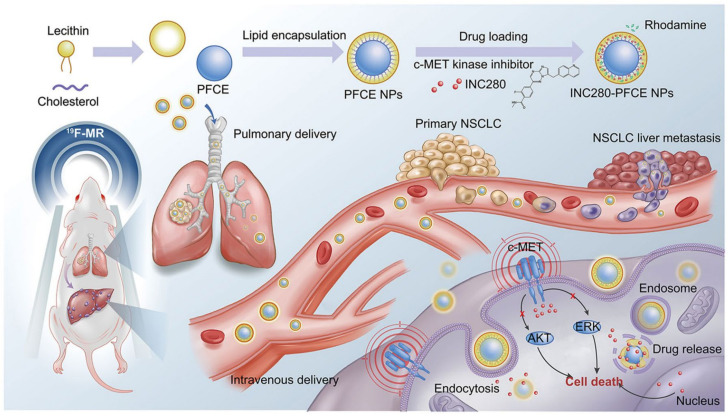
The scheme of the synthesis of the INC280-PFCE NPs and theranostic strategy [37]. Reprinted with permission from *ACS Nano* **2022**, *16*, 12590–12605. Copyright 2022, American Chemical Society.

With relatively long and stable bioretention properties and extremely favorable ^19^F-MRI properties, perfluoro-15-crown-5-ether (PFCE) provides an opportunity to build a more effective image-guided therapeutic platform to precisely treat cancer. Recently, two PFCE-based delivery systems containing EGFR-TKI AZD9291 [37] or MET inhibitor INC280 [38] have been reported, respectively. In detail, the PFCE acts as the core, with a lipid monolayer formed by cholesterol and lecithin as the shell. In addition to improving drug loading efficiency through the host–guest interactions, PFCE can also mediate ^19^F-MRI-guided low-intensity focused ultrasound. Therefore, the PFCE delivery system containing INC280 could achieve synergistic treatment and integrated diagnoses for primary NSCLC and its liver metastases (Figure 5) [37].

## 3. Cyclodextrin-Based DDSs

**Figure 6:**
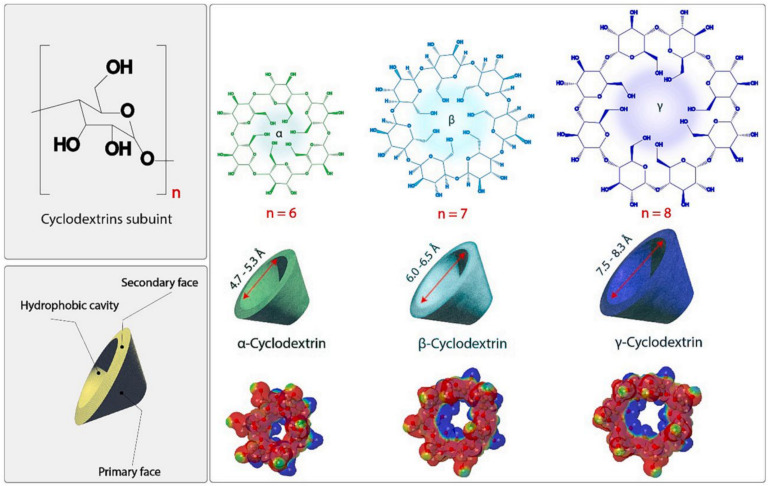
Chemical structures of α-, β-, and γ-CD [40]. Reprinted with permission from *Carbohydr. Polym.* **2024**, *324*, 121500. Copyright 2024, Elsevier.

Cyclodextrins (CDs) are a class of cyclic oligosaccharides linked by α-1, 4-glucoside bonds with d-glucose units [39,40]. The most commonly used CDs are α-, β-, and γ-CD with six, seven, and eight glycosyl units, respectively (Figure 6), and their derivatives. The number of repetitions of glucose units determines the size of cavities and physical properties of CDs. The cavity of CD is hydrophobic, while the outside is hydrophilic due to the presence of hydroxyl groups. Thus, CDs are usually combined with hydrophobic substances to form host–guest complexes (mostly at a 1:1 ratio) [41]. CDs can be used independently to achieve solubilization and controlled release, attenuating toxicity and improving bioavailability of drugs. Additionally, CDs widely participate in the construction of almost all NDDSs, including liposomes, emulsions, micelles, etc., and play their unique roles in multistimuli-responsive drug release [42,43]. Recent studies have focused on the constructions of CD-based micro- or nanostructures, which endowed them with specific functions. In general, these nanostructures are intentionally prepared based on three important characteristics of CDs: (1) pre-organized three-dimensional molecular structure; (2) easy to chemically modify to introduce functional groups; (3) forming dynamic inclusion compounds with various guests in an aqueous solvent.

### 3.1. CD-Based Liposomes/Niosomes

Liposomes/niosomes have been widely used in drug delivery [44,45]. However, lipophilic drugs can only be loaded into the thin lipid layer of ordinary liposomes through hydrophobic interactions, which results in low loading content and accidental drug leakage in the body. CD-based liposomes/niosomes, which cover above shortages, were designed to improve the encapsulation efficiency of insoluble drugs [46].

Slightly soluble drugs can be loaded into the aqueous core of liposomes in the form of inclusion complexes with CDs, which greatly enhances the drug loading efficiency [47,48]. This simple strategy is applicable to a variety of pharmaceutical drugs, including steroidal or non-steroidal anti-inflammatory drugs, anti-tumor drugs, antibacterial drugs, etc. [49]. For instance, anti-glaucoma drug brinzolamide forming inclusion complexes with hydroxypropyl-β-CD (HP-β-CD) can be loaded into liposomes with an extremely high drug encapsulation efficiency (92.5%) [50]. Moreover, this new formulation exhibited a sustained release property compared to liposomes directly loaded with prototype drugs. Another study reported a similar result of a liposome loaded with a HP-β-CD–ropivacaine complex [51]. Compared with free drugs and liposomal drugs, this ternary DDS containing HP-β-CD showed a better anesthetic effect: an in vivo sensory blockade effect increased by 1.7 times and 1.3 times, respectively. Further, this HP-β-CD-based liposome significantly reduced the toxicity of ropivacaine to 3T3 fibroblasts [51].

In addition to classical liposomes, CDs can also be involved in the construction process of multivesicular liposomes (MVLs) [52]. For example, celecoxib was prepared into an inclusion compound with β-CD, and then multivesicular liposomes loaded with drugs were prepared by reverse phase evaporation [53]. The results confirmed that this β-CD-based MVL exhibited a very high drug loading (88%). The drug release time has significantly increased, with the time required to release over 80% of the drug increasing from 48 h to 120 h. Most importantly, the intraperitoneal injection of MVLs showed more persistent and superior anti-inflammatory activity in alleviating a carrageenan-induced rat toe swelling model [53]. Meanwhile, the performances of MVL formulations mediated by different types of CDs were also compared. For fluoroacetone, the HP-β-CD-based MVLs provided the optimal drug loading, sustained release ability, and efficacy for ocular inflammatory diseases [54].

Further, CDs can also participate in the design and preparation of deformable liposomes. The first CD-based deformable liposome was used to achieve transdermal delivery of meloxicam [55]. The solubilization effect of β-CD was favorable for the permeation rate of meloxicam. Additionally, the loading content of CD-based deformable liposomes is 1.4 and 9.1 times higher than that of corresponding CD-free meloxicam-loading liposomes and simple suspension, respectively.

Niosomes are non-ionic surfactant-based structures formed by self-assembly in aqueous media of non-ionic amphiphilic molecules, resulting in bilayer vesicles [56]. Compared with liposomes, niosomes are believed to have lower cost, better stability, higher fluidity, and better modifiability [57]. They have been proposed as an alternative to liposomes as drug carriers. A series of CD-based niosomes were also successfully developed [46,58,59]. For instance, the Span 80/Tween 80 niosomes in the presence of β-CD improved the amount of an entrapped hydrophilic molecular probe (methyl orange) and produced a faster dye release. However, in the presence of a modified amphiphilic β-CD (Mod-β-CD), the niosomes became smaller. This may be a result from the anchoring of Mod-β-CD at the surface of vesicles through the hydrophobic chain, altering the curvature of the outer monolayer and reducing the surface charge of the interphase [58]. Another example is that an ibuprofen–β-CD complex was encapsulated in Tween 20/cholesterol vesicles to form a new niosome (ibuprofen–βCd–NSV). Compared with plain drug suspension, ibuprofen–βCd–NSV exhibited improved drug permeation properties and therapeutic efficacy [59].

A very noteworthy issue is that although CDs can increase the drug loading capacity of lipomes/niosomes on insoluble drugs, its natural encapsulation effect on lipids (regardless of whether CDs exist in an aqueous core or lipid layer) may affect the fluidity of lipid membranes or lead to a decrease in their stability.

### 3.2. CD-Based Polymeric NPs

The utilization of biodegradable natural or synthetic polymers as drug carriers has been well documented to have several important advantages: endowing drugs better stability, longer half-lives, higher drug loads, more diverse delivery routes, and more controlled (on-demand or targeted) release properties [3]. Further, the introduction of CDs into polymer nanoparticles (NPs) will facilitate the integration of the above-mentioned advantages into NDDSs [60,61].

Chitosan (CS) is one of the most commonly used natural polymers in combination with CDs. Since CS is a cationic polymer, the construction process of CS NPs is usually by adding a negative electric coupling agent (such as tripolyphosphate) to an aqueous medium containing CS, thus avoiding the use of organic solvents [62]. However, this preparation method strongly limits the embedding ability of CS NPs towards hydrophobic drugs [63]. As early as 2006, researchers proposed the combined application of CDs and CS. Indeed, it has been experimentally verified that the drug loading capacity of CS NPs is significantly increased by using HP-β-CD–triclosan or furosemide as the payloads [64]. Another similar strategy reported is the direct action of anionic carboxymethyl-β-CD (CM-β-CD) as a cross-linker mediating the formation of CS NPs, while solving the dissolution problem and encapsulation efficiency of the non-steroidal anti-inflammatory drug sulindac [65]. Sulfobutylether-β-CD (SBE-β-CD)-based CS NPs can also use the same strategy to achieve high-efficiency loading of steroidal anti-inflammatory drugs hydrocortisone [66], Ibrutinib [67], naringenin [68], and idebenone [69]. It is logical that CD-based CS NPs could also achieve co-delivery of hydrophobic and hydrophilic drugs. For example, β-CD and HP-β-CD can form inclusion complexes with methotrexate, respectively, and both can be loaded into CS NPs along with water-soluble drug calcium folinate [70]. Generally speaking, CD-based CS NPs could improve drug encapsulation efficiency, meanwhile achieving drug sustained release [70].

Another strategy is preparing CD-grafted CS, a polymer derivative with inclusion capability, which can also achieve high drug loading. For example, NPs based on HP-β-CD-grafted CS have stronger intestinal permeability and absorption ability. It has been proven that HP-β-CD can help the system open the tight junctions and enhance the clathrin-dependent endocytosis, macro-pinocytosis, and phase of the essential epithelial cells [71].

In addition to CS, other CD-based natural polymers and synthetic polymers have also been developed to design NDDSs. The natural polymers include alginates, hyaluronic acid, glucans, etc. [72], and the synthetic polymers include polyethyleneimine (PEI), polycaprolactone (PCL), polylactic acid (PLA), polyglycolic acid (PGA), poly(lactic-co-glycolic acid) (PLGA), etc. [73]. Among them, the FDA-approved PLGA is considered to have excellent biodegradability and biocompatibility [74]. The common strategies to introduce CDs into PLGA are encapsulating CD–drug complexes [75,76,77] in PLGA NPs or preparing CD-modified PLGA derivatives [78,79,80]. It is gratifying that CD-based PLGA derivatives have improved loading efficiency, sustained release effect, and corresponding therapeutic effects of drugs.

**Figure 7:**
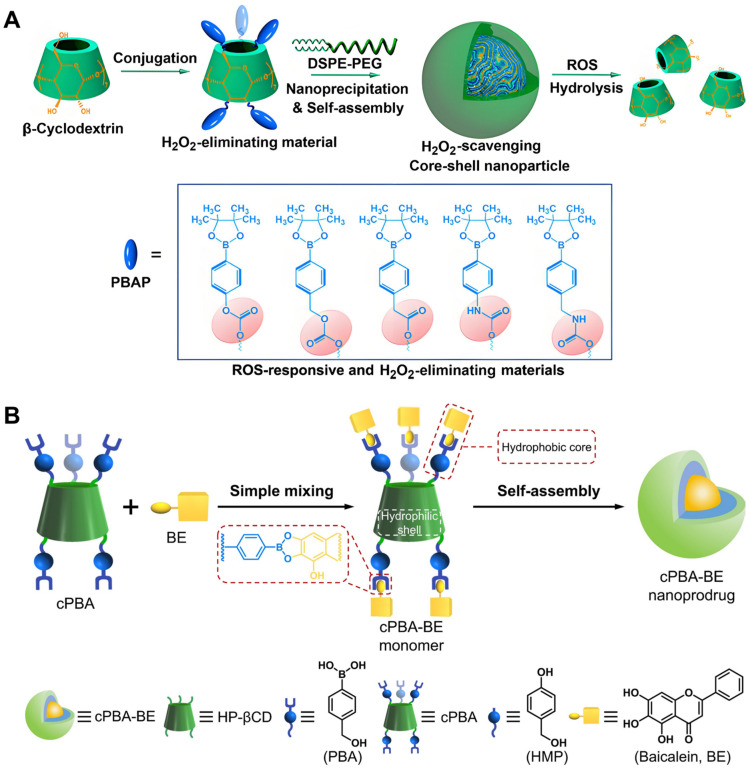
NPs constructed directly using β-CD derivatives. (**A**), ROS-responsive and H_2_O_2_-eliminating core–shell NPs were prepared by grafting phenylboronic acid pinacol ester derivatives (PBAP) onto β-CD [81]. Reprinted with permission from *Chem. Mater.* **2017**, *29*, 8221–8238. Copyright 2017, American Chemical Society. (**B**), A cyclic phenylboronic acid (cPBA) based on β-CD was synthesized, and an inflammatory microenvironment (ROS and acidic environment)-responsive baicalin nanoprodrug was constructed by a dynamic phenylborate bond [85]. Reprinted with permission from *Theranostics* **2021**, *11*, 8301–8321. Copyright 2021, Ivyspring International Publisher.

Drug NPs could be constructed directly from CDs without the polymers. In Figure 7A, a series of ROS responsive and scavenging functional materials were constructed by coupling various derivatives of phenylboronic acid pinacol ester onto β-CD, and corresponding nanoparticles were formed through self-assembly [81]. By utilizing the hydrophobic core and the cavity of β-CD, free radical scavengers or anti-tumor drugs can be loaded to effectively treat inflammation-related diseases in mice, such as colitis [82], colitis-related colon cancer [83], and balloon injury-induced restenosis [84]. A gripper-like phenylboronic acid-coupled β-CD was also developed, and a nanoprodrug loaded with baicalin (an *o*-diol drug) was prepared by borate ester bond formation, which was successfully applied to alleviate drug-induced hepatitis in mice (Figure 7B) [85].

### 3.3. CD-Based Lipid NPs

Compared to traditional liposomes, new lipid NPs represented by solid lipid NPs (SLNs) [86,87] and nanostructured lipid carriers (NLCs) [88,89] exhibit better physical stability and good permeability. Many studies have shown that the strategy of introducing CDs can improve the multifaceted capacity of lipid NPs. For example, a paclitaxel-loaded SLN [90] was developed using a strategy of incorporating CD–drug inclusion compounds into SLNs. Specifically, the AUC_0→∞_ of paclitaxel-loaded SLNs containing HP-β-CD in rats was 2.0 and 1.5 times higher than that of the drug solution and SLNs without HP-β-CD, while t_1/2_ was 2.3 and 1.5 times higher, respectively. And then stronger anti-tumor activity and lower nephrotoxicity were ultimately achieved [90]. Another research group developed a new type of oral hydrochlorothiazide formulation. Compared with SLNs without HP-β-CD, HP-β-CD-based SLNs exhibited a higher drug encapsulation efficiency (from 37.5% to 66.5%) and drug release rate [91]. The same strategy was applied to the NLCs loaded with hydrochlorothiazide [92,93]. HP-β-CD endows NLCs with a higher encapsulation efficiency and faster release rate for drugs (100% released at 6 h, vs. 60%). According to this, lipid NPs containing HP-β-CD achieved a stronger diuretic effect and maintenance time in animal models. As expected, HP-β-CD has played a good role in improving the preparation of drug-loaded lipid NPs such as irbesartan [94], volatile essential oil [95], and other active ingredients from plant sources [96].

**Figure 8:**
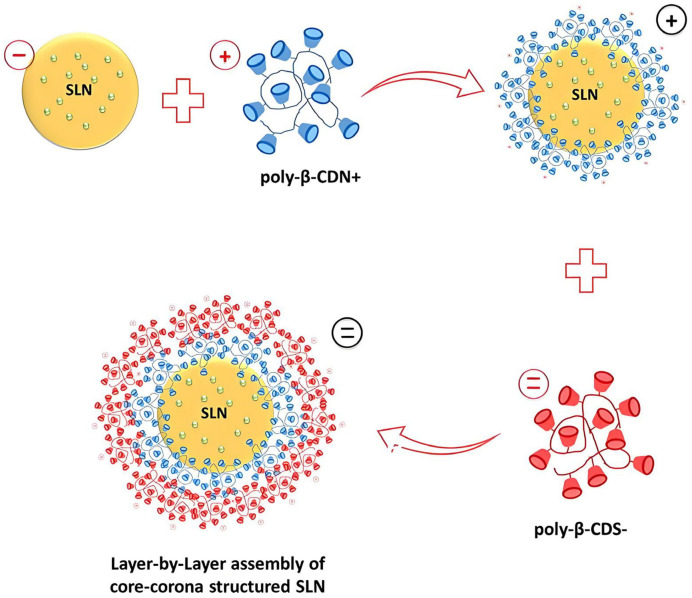
Layer-by-layer coating process to create core–corona-structured solid lipid NPs. Oppositely charged β-CD polymers were employed for surface modification [97]. Reprinted with permission from *Int. J. Pharm.* **2021**, *592*, 119994. Copyright 2021, Elsevier.

A layer-by-layer assembly core–corona nanoarchitecture has also been reported, which is constructed of poly-β-CD and SLNs with two opposite charges (Figure 8). The layer based on poly-β-CD with opposite charges effectively transitions the zeta potential of SLNs from negative to positive, and the surface charge density significantly increases compared to the core SLNs. The results indicated that poly-β-CD can enhance the sustained/controlled release of drugs [97].

### 3.4. CD-Based Emulsions

Micro-emulsions (MEs) and nano-emulsions (NEs) are isotropic, transparent, and thermodynamically stable colloidal systems consisting of oil and water phases stabilized by the presence of surfactant or cosurfactant molecules. The main reason for using CDs in combination with MEs or NEs is to further improve the performance of MEs/NEs. CDs could act as emulsifiers and stabilizers to prepare Pickering emulsion [98,99,100,101]. Research has shown that using β-CD or β-CD–cinnamaldehyde inclusion complexes as emulsifiers in the preparation process of various Pickering emulsions with different oil phases can significantly improve the stability of Pickering emulsions in the range of 20~65 °C [98].

Other studies have found that CD–drug inclusion compounds make drug-carrying NEs have a more controlled and slower release characteristic. Compared to the CD-free drug-carrying NEs, HP-β-CD–resveratrol inclusion compound-loaded NEs showed better ability to prevent drug degradation under long distance UV exposure (degradation rate decreased from 57.6% to 25.2%) [102]. The same strategy was also applied to MEs loaded with HP-β-CD–apigenin inclusion complexes [103]. Further studies proved that CD could improve the skin penetration of emulsion. It was observed in CryoTEM that adding α-, β-, and γ-CD, respectively, seemed to alter the microstructure of sucrose stearate-based or lecithin-based NEs [104,105,106]. Experiments showed that these CD-based NEs had better colloidal stability and permeability to pig skin (55.10 vs. 9.99 µg/cm^2^) [104], and correspondingly improved the biological effect of fludrocortisone acetate [105].

### 3.5. CD-Based Micelles

Due to the ability to dissolve lipophilic drugs in aqueous solutions and improve their bioavailability, micelles have been widely used as drug carriers. However, the poor solubilization effect, high critical micelle concentration (CMC), and potential adverse reactions after intravenous injection have limited the clinical application of surfactant micelles [107]. Polymeric micelles offer greater advantages over conventional surfactant micelles in terms of solubilization capacity, lower CMC, higher stability, and better tolerance [108].

**Figure 9:**
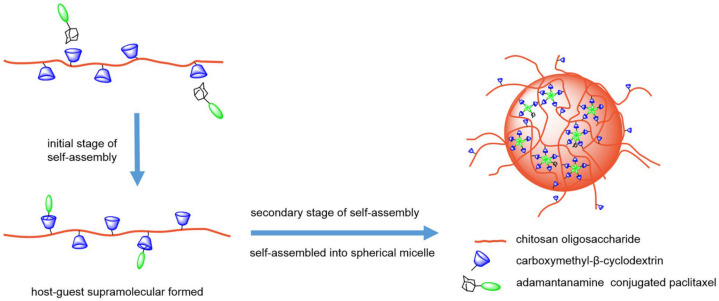
CM-β-CD is grafted to a chitosan oligosaccharide to form a hydrophilic polymer, and the synthesized amantadine–paclitaxel conjugates enter the CM-β-CD cavity as hydrophobic groups, and then self-assemble to form micelles [110]. Reprinted with permission from *PLoS ONE* **2016**, *11*, e0150877. Copyright 2016, Public Library of Science.

The strategy of combining CD encapsulation and micelle loading has been well explored [109]. As shown in Figure 9, the amantadine–paclitaxel conjugate, as a guest, interacts with CM-β-CD on the chitosan oligosaccharide. Thus, a novel micelle CSO-*g*-CM-β-CD@AD-PTX was successfully prepared [110]. The results show that the supramolecular micelle has spherical core–shell structure with good colloidal stability. The drug loading content is up to 31.1% and CMC is only 3.4 × 10^−7^ M. In addition, CSO-*g*-CM-β-CD@AD-PTX showed excellent sustained release ability; only 63.1% of AD-PTX was released from the micelle within 30 days.

It is worth noting that CD-based micelles can also load nucleic acids for gene therapy. For example, β-CD-grafted polyetherimide derivatives are coupled with folic acid to form tumor-targeting micelles (β-CD-PEI-FA). Experimental data proved that the biocompatible nanocarriers could efficiently encapsulate miRNA, resist degradation by a serum and nuclease, inhibit the expression of Kaposi’s sarcoma-associated herpesvirus, and thus inhibit tumor development [111].

### 3.6. CD-Based Hydrogels

Hydrogels with porous cross-linked 3D networks have received much attention in drug delivery applications due to their ability to improve the bioavailability and solubility of hydrophobic drugs [5]. CDs not only enhance the solubility of drugs, but also allow polymers to pass through the cavities, which are considered as cross-linking agents [112,113].

It has been reported that PEG and α-CD could form stable hydrogels. More importantly, the gel–solution transition temperature of CD-based hydrogel can be adjusted according to the length of the polymer chain and the concentration of CDs to achieve personalized customization [112]. Researchers have developed an injectable hydrogel with α-CD and 4-arm PEG as raw materials. α-CD increased the solubility of the drug by nearly 50%, and the cell viability did not show toxicity at a higher dose of hydrogel [114]. In addition, a double-layer hydrogel was prepared with poly-β-CD, polyvinyl alcohol, and sodium carboxymethyl cellulose as raw materials. The hydrogel provides a feasible scheme of high-performance mechanical dressing with drug sustained release [115].

The above examples demonstrate the unique values of introducing CDs in almost all types of DDS design processes.

## 4. Cucurbit[n]uril-Based DDSs

**Figure 10:**
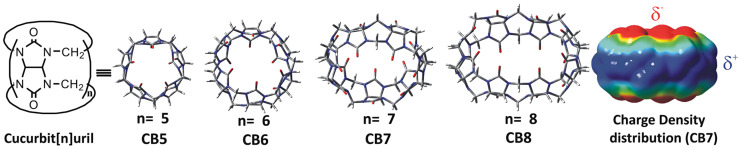
Geometry-optimized structures of CB[n]s: CB[5], CB[6], CB[7], and CB[8]. The calculated charge density distribution in CB[7] [119]. Reprinted with permission from *Langmuir* **2022**, *38*, 6249–6264. Copyright 2022, American Chemical Society.

Cucurbit[n]urils (CB[n]s, n = 5~8, 10, or 13~15) are cyclic copolymers with an internal hydrophobic cavity and polar carbonyl groups. This kind of rigid host molecule is similar to pumpkin (Figure 10). Although the cavity size of CB[n]s and CDs is similar, the properties are not the same. A large number of studies have shown that the binding of guest molecules with CDs mainly relies on the hydrophobic force [116]. The hydroxyl groups at both edges of the CD cavity face outward, rarely generating strong interactions with guest molecules. However, the binding of guest molecules to CB[n]s mainly depends on two forces: (1) the ionic–dipole interaction of the positive charge from guest molecules and the carbonyl oxygens from CB[n]s; (2) hydrophobic interaction between the guest and host [117]. Additionally, the polar carbonyl oxygens of CB[n]s can also have strong interaction with metal cations [118]. Therefore, CB[n]s have been proved to prefer to form complexes with protonated alkyl compounds or aromatic amines with high binding affinity [119]. In the field of DDSs, CB[n]s are generally regarded as controlled release carriers, detoxifying carriers, and targeted delivery carriers of drugs or genes. They can also be assembled into biomacromolecular assemblies and show tremendous application potential in bio-sensing and disease treatments [120]. However, the relatively poor solubility of most CB[n]s and the structure are not easy to modify, greatly limiting its further development in applications.

### 4.1. CB[n]-Based Inclusion Complexes

CB[n]s with hydrophilic surfaces and hydrophobic cavities can form inclusion complexes with lipophilic drugs to improve their solubility and stability. CB[5] always complexed with small molecules and was used as an ion container [121,122], because of its limited cavity size. CB[7] is most widely used because of its suitable cavity size, excellent water solubility, and low toxicity [123]. For example, CB[7] has been reported to directly incorporate triamterene to improve its stability [124]. CB[7] increased the water solubility of cholesterol β-estradiol [125]. The inclusion complex based on CB[7] increased the uptake of nitidine chloride by tumor cell line MCF-7 cells, while it decreased it in liver cell line L02 cells, resulting in reduced toxicity and increased efficiency [126]. Compared to CB[7], CB[8] is large enough to accommodate two guests in the cavity at the same time [127], thus providing a good platform for synergistic therapies.

**Figure 11:**
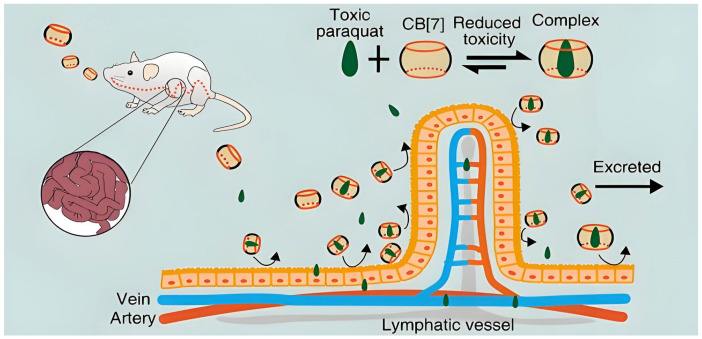
CB[7] used as a specific oral antidote for paraquat poisoning by strongly binding with paraquat and inhibiting its absorption in the gastrointestinal tracts [128]. Reprinted with permission from *Theranostics* **2019**, *9*, 633–645. Copyright 2019, Ivyspring International Publisher.

CB[n]s can also be considered as antidotes to avoid or reduce the toxic side effects of guest molecules. CB[7] was shown to significantly alleviate the toxicity of paraquat by reducing its concentration in plasma and major organs, thereby reducing mortality and associated adverse reactions in paraquat-poisoned mice (Figure 11). The experimental results show that CB[7] is more effective than the “gold standard” detoxifying agent activated carbon for treating paraquat poisoning [128]. Similarly, CB[7] can also serve as an antidote to neuromuscular blockers. Succinylcholine is the only widely used depolarizing neuromuscular blocker in emergency care, but it has serious side effects. CB[7] could reduce the toxicity of succinylcholine through host–guest encapsulation [129]. Bedaquiline is an anti-tuberculosis drug with cardiotoxicity and poor water solubility. By encapsulation with CB[7], the water solubility of bedaquiline in acidic and neutral media increased. In vitro and in vivo experimental data showed that the cardiotoxicity of bedaquiline decreased, while its anti-mycobacterial activity remained unchanged [130]. Another example is that arecoline hydrochloride (AH) exhibits severe hepatotoxicity while treating for several neurological diseases. It was found that AH and CB[7] formed a complex with high binding affinity, and significantly reduced the liver toxicity of AH in vitro [131]. All these experiments show that CB[n]s can be excellent supramolecular antidotes.

It is worth mentioning that the continuous epidemic outbreak of RNA virus-induced disease in the population highlights the need to develop broad-spectrum drugs against RNA viruses. Polyamines (including putrescine, spermidine, and spermine) can serve as potential targets. This is because polyamines are positively charged metabolites in host cells and play important roles in RNA virus replication [132,133]. Excitingly, CB[7] can capture spermine molecules to reduce its intracellular level and block the replication process of RNA viruses (including enteroviruses, flaviviruses, alphaviruses, etc.) [134]. Therefore, CB[7] is considered to have broad-spectrum anti-RNA virus activity.

### 4.2. CB[n]-Based NDDSs

**Figure 12:**
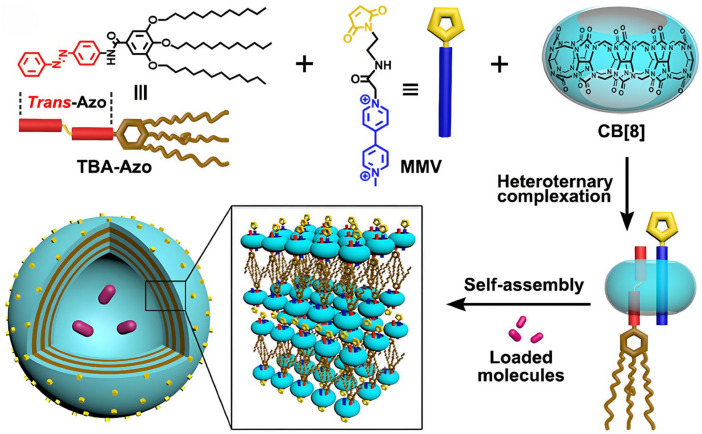
Schematic diagram of construct supramolecular vesicles by photo-responsive heteroternary complexation-based CB[8], TBA-Azo, and MMV [135]. Reprinted with permission from *ACS Appl. Mater. Interfaces* **2018**, *10*, 4603–4613. Copyright 2018, American Chemical Society.

In addition to acting as macrocyclic carriers to directly load specific guest molecules, CB[n]s can also be involved in the construction of other types of NDDSs. The complexation behavior of CB[n]s with certain moieties can be utilized to prepare amphiphilic compounds, then induce self-assembly of nanostructures. For example, researchers have utilized CB[8] to simultaneously encapsulate methylviologen (MMV) and a 3,4,5-tris-(n-dodecyloxy)-benzoylamide-azobenzene conjugate (TBA-Azo) (Figure 12). The heteroternary supramolecular vesicles were formed by self-assembly of CB[8] with the inclusion complexes of MMV and AZO as hydrophilic components and TBA as hydrophobic components [135]. DOX can be further loaded into the core of the vesicles. UV irradiation can induce the change in TBA-Azo configuration from *trans* to *cis*, thereby inducing the dissociation of supramolecule vesicles and the subsequent release of DOX. That confirms that this kind of supramolecule vesicle is an excellent UV-sensitive drug carrier [135].

Moreover, CB[n]s can also act as cross-linking agents to participate in the formation of hydrogels. For instance, researchers have explored a hydrogel based on CB[8] and phenylalanine-grafted chitosan. CB[8] acts as a cross-linking agent to construct a 3D network structure by including two phenylalanine units into its cavity. During the preparation of the hydrogel, anticancer drugs can be loaded into the gel matrix [136]. There is another study proving that CB[7] can reduce the non-specific cytotoxicity of branched PEI (bPEI) through supramolecular wrapping of the bPEI by CB[7] [137]. More importantly, this strategy does not affect the cell uptake and gene transfection efficiency mediated by bPEI [137]. Therefore, CB[n]-based delivery systems can also serve as an effective gene delivery strategy.

### 4.3. Acyclic CB[n]-Based DDSs

Acyclic CB[n] does not have a closed circular structure, which endows them special physical and chemical properties [138,139]. Due to the larger, more irregular, clip-like shape of the cavity, acyclic CB[n]s can bind to larger poorly soluble drugs to enhance their solubility and bioactivity [140]. It has been proved that acyclic CB[n]s increased the solubility of insoluble drugs by a factor of between 23 and 2750 by forming container–drug complexes [140]. Especially for paclitaxel, acyclic CB[n]s exhibit far superior solubilization ability (2750-fold) than that of HP-β-CD. The increased concentrations of paclitaxel ultimately resulted in more efficient killing of HeLa and SK-OV-3 cancer cells than paclitaxel alone [140].

The study confirmed the inclusion effect of two types of acyclic CB[n]s (calabadion 1 and calabadion 2) on multiple nonopioid drugs (methamphetamine, fentanyl, cocaine, ketamine, phencyclidine, morphine, and hydromorphone) [141]. Compared to other macrocyclic molecules (CB[7], C[4]As, and HP-β-CD), calabadion 2 exhibits good binding affinity for methamphetamine and can reverse the high motor ability induced by methamphetamine in rats [141]. A study of acyclic CB[n]s binding with neuromuscular blocking agents (NMBAs) and reversing a neuromuscular block was also conducted. Because of the outstanding affinity, acyclic CB[n] was able to significantly reverse a deep rocuronium-induced neuromuscular block in rats [142].

In addition to being directly used as inclusion agents, acyclic CB[n]s were also utilized to build more diversified NDDSs in recent years. For instance, acyclic CB[n]-based nanosponges, which could improve the cell uptake efficiency, can be fabricated via supramolecular vesicle-templated cross-linking. When photodynamic therapeutic (PDT) drug temoporfin was loaded, the PDT efficacy was greatly enhanced for cancer cells [143]. Moreover, acyclic CB[n] can be modified to biotin–acyclic CB[n], and then form an amphiphilic complex with amantadine-conjugated cannabinoids. A cell-targeted supramolecular micelle was further obtained through amphiphilic self-assembly to achieve tumor-targeted delivery of cannabidiol [144].

### 4.4. CB[n]-Based Supramolecular–Organic Frameworks

**Figure 13:**
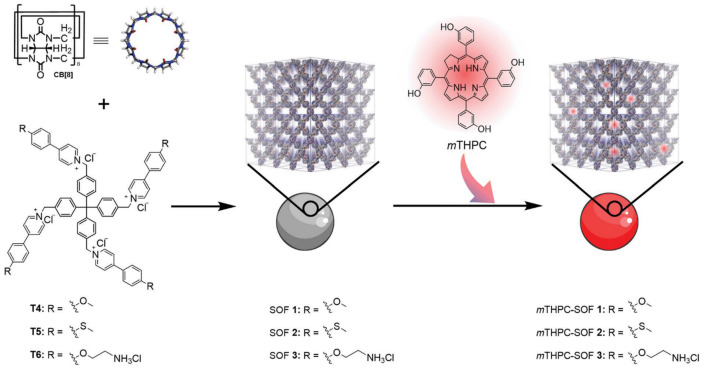
A schematic illustration of the chemical structures of SOF monomers (CB[8] and T4−6) and photodynamic drug *m*THPC, and the construction of CB[8]-based SOFs [146]. Reprinted with permission from *J. Mater. Chem. B* **2022**, *10*, 899–908. Copyright 2022, Royal Society of Chemistry.

Recently, CB[n]s were used to construct 3D homogeneous supramolecular organic frameworks (SOFs) as NDDSs. As porous biomaterials with hydrophobic apertures, water-soluble 3D SOFs show great potential for self-assembly with hydrophobic photodynamic therapeutic agents or insoluble drugs. For example, SOFs with nano-scaled pores could be constructed by the encapsulation of aromatic dimerization by CB[8]. Hydrophobic drug DOX was further adsorbed, driven by hydrophobicity. The DOX-loaded SOFs exhibited pH-responsive release properties and enhanced anti-tumor efficacy [145]. This new delivery strategy offers advantages over conventional DDSs. Specifically, CB[8]-based SOFs omit the drug loading procedures and avoid the introducing of any stimuli-responsive motif for controlled release. Other studies used CB[8]-based SOFs to deliver porphyrin photodynamic agents (Figure 13), indicating that SOFs were able to enhance the PDT efficacy through reducing the aggregation-caused quenching effect [146], meanwhile suppressing sunlight-induced skin phototoxicity [147].

## 5. Calix[n]arene-Based DDSs

**Figure 14:**
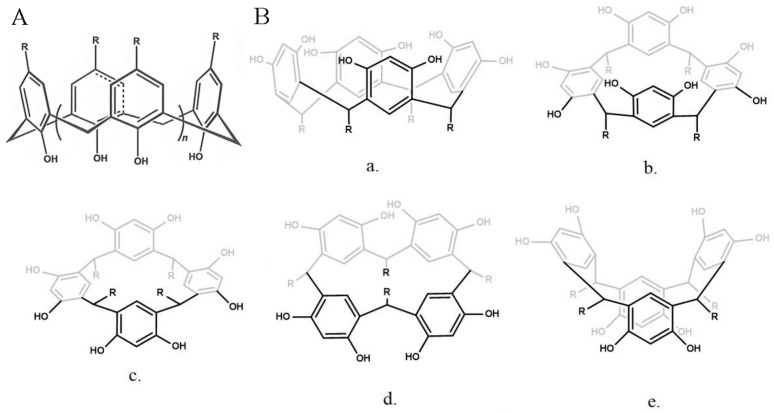
(**A**) Chemical structure of C[n]As, (**B**) conformations of calix[4]resorcinarenes: (**a**) crown; (**b**) boat; (**c**) chair; (**d**) diamond; (**e**) saddle [151]. Reprinted with permission from *J. Nanomed. Res.* **2015**, *2*, 28. Copyright 2015, MedCrave Group.

As shown in Figure 14A, calix[n]arenes (C[n]As) are macrocycles composed of methylene-bridged phenol units, with a structure similar to a cup, known as the third-generation host compound [148]. C[n]As and its water-soluble derivatives have good biocompatibility and low cytotoxicity. Compared with CEs, CDs, and CBs, the functionalization of C[n]As is easier to control. C[n]A is able to bind with a large variety of guest molecules, including metal ions, amino acids, alkyl derivatives, etc., to form different aggregations [149]. Besides the inclusion occurring inside the cavity of C[n]As, some proteins and nucleic acids could also bind at the edges of C[n]As then modulate the bioactivity [150]. Resorcinarenes are another kind of attractive macrocyclic molecule for drug delivery. The commonly used calix[4]resorcinarenes consist of four resorcinol units linked by methylene bridges, enabling the formation of five distinct conformations (Figure 14B) [151]. These configurations facilitate the formation of stable host–guest complexes, rendering them great potential as drug carriers.

### 5.1. C[n]A-Based Inclusion Complexes

**Figure 15:**
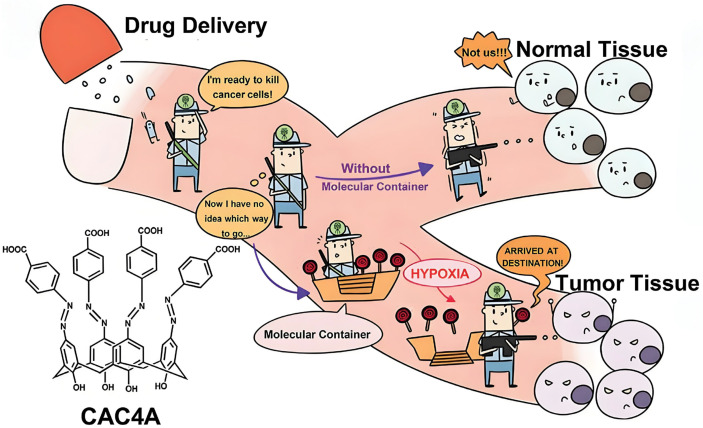
A schematic illustration of the hypoxia-responsive drug delivery mechanism of CAC[4]A [152]. Reprinted with permission from *Adv. Mater.* **2020**, *32*, e1908435. Copyright 2020, Wiley.

The poor water solubility and toxicity limit the application of C[n]As in the biomedical field. Thus, C[n]A derivatives, which were synthesized by modification on the upper and lower edges of C[n]As, were widely used to overcome these shortcomings [148]. For example, a hypoxia-responsive molecular container based on carboxylated azocalix[4]arene (CAC[4]A) was prepared and used in cancer therapy (Figure 15). CAC[4]A showed strong host–guest recognition ability for 12 chemotherapeutic drugs, indicating its good universality [152]. Recently, in order to develop an artificial receptor with high binding affinity, a naphthyl azo-unit-modified azocalix[4]arene (Naph-SAC4A) was synthesized. Naph-SAC4A effectively expands the hydrophobic cavity surface of AC[4]A, resulting in remarkable enhancement in binding affinity to an outstanding level of 10^13^ M^−1^. Moreover, the hypoxia-cleavable azo-bonds in Naph-SAC4A impart controllable release of DOX at the cellular level [153]. C[n]A derivatives can not only incorporate small-molecule drugs, but also be used to carry nucleic acid drugs. Lomazzi prepared tetra-L-argino-tetrahexyloxy-C[4]A and confirmed its extraordinary ability to compact and internalize different types of nucleic acid cargos (DNA, miRNA, PNA) into cells [154].

### 5.2. C[n]A-Based NDDSs

Like other macrocycles, C[n]As also facilitates the construction of different types of supramolecular assemblies, such as micelles, vesicles, and nanoparticles. These characteristics make it a research hotspot in the field of cancer treatment. For example, phosphorylated C[4]As can co-encapsulate camptothecin and paclitaxel and further form nanovesicles, leading to maximal tumor growth inhibition and higher efficacy [155]. The PEGylated tert-butyl C[4]A or tert-octyl C[8]A can self-assemble into micelles and greatly improve the solubility of curcumin [156] and silybin [157], respectively.

Furthermore, an amphiphilic C[5]A-based nanovesicle (named CASTING) that loaded STING agonists was used as a nano-vaccine [158]. CASTING can not only improve the transmembrane transport efficiency and immunostimulatory activity of CDGSF (a chemically modified cyclic di-GMP (CDG)) in cells, and reduce immune-related side effects, but also induce the formation of an immunogenic microenvironment in tumor-bearing mice. A polymer nanoparticle (PHEMA-SAC[4]A) empowered by sulfonated azocalix[4]arene (SAC[4]A) has achieved in vivo targeted drug delivery and drug tracking [159]. SAC[4]A exhibits high affinity for various anticancer drugs under normoxic conditions, while exhibiting poor affinity under hypoxic conditions. This property enables PHEMA-SAC[4]A to effectively achieve passive targeted drug release in tumor hypoxic environments.

C[n]As have also been reported to be used in the development of gene delivery therapy systems. Amphiphilic C[4]A derivatives were developed by modifying C[4]A with three aminoglycosides, all of which have higher affinity with DNA than PEI. All three C[4]A nanoassemblies formed with DNA showed excellent transfection efficiency and negligible cytotoxicity in both Hela and U87-MG cells [160].

In addition, hydrogels can also be constructed based on the hydrophobic cavities of C[n]A derivatives. Bandela et al. synthesized cholesterol-modified C[4]A. This amphiphilic C[4]A derivative can repeat self-inclusion to form a cross-linked hydrogel network, so as to load drugs including DOX, curcumin, tocopherol, etc., and quickly release drugs after simple heating [161]. Other researchers have developed a hydrogel based on methacrylated SAC[4]A (SAC[4]A-MA), methacrylated hyaluronic acid (HA-MA), and dithiol-terminated matrix metaproteinase 13-sensitive peptide [162]. The hydrogel loaded anti-inflammatory drug hydroxychloroquine through host–guest interactions, and successfully treated the rat osteoarthritis model by local injection.

### 5.3. Calix[4]resorcinarene-Based DDSs

Calix(4)resorcinarene-based DDSs were always constructed for the controlled release and sustained release of hydrophobic drugs. For instance, an amphiphilic multi-tailed resorcinarene (MTR) was synthesized by modifying calix(4)resorcinarene with 4-hydroxybenzaldehyde. MTR can self-assemble into vesicles in an aqueous medium to entrap hydrophobic drugs (such as clarithromycin), with an entrapment rate of 65.12 ± 3.31%. The drug can be released for more than 20 h [163].

Recently, a series of stimulus-responsive DDSs based on calix[4]resorcinarenes have been developed. For example, calix[4]resorcinarenes and methoxy-PEG were conjugated via acylhydrazone bonds to form calix[4]resorcinarene-mPEG, a pH-sensitive and low-toxic conjugate. When arriving in the acidic tumor microenvironment (pH 5.5), conjugate-encapsulated DOX or methylene blue (MB) hydrolyzes and the drug releases, resulting in enhanced efficacy against tumor cells or improved PDT effects, respectively [164]. Similarly, Sergeeva et al. constructed a pH-responsive polymer nanocarrier based on calix[4]resorcinarenes for DOX delivery by covalently linking *N*-methyl-d-glucamine resorcinarenes with phenylboronic acid [165]. DOX-loaded nanoparticles remained stable at normal pH, but hydrolyzed and released the drug at pH below 6. Therefore, constructing supramolecular pH-responsive DDSs based on calix[4]resorcinarenes represents a promising strategy.

**Figure 16:**
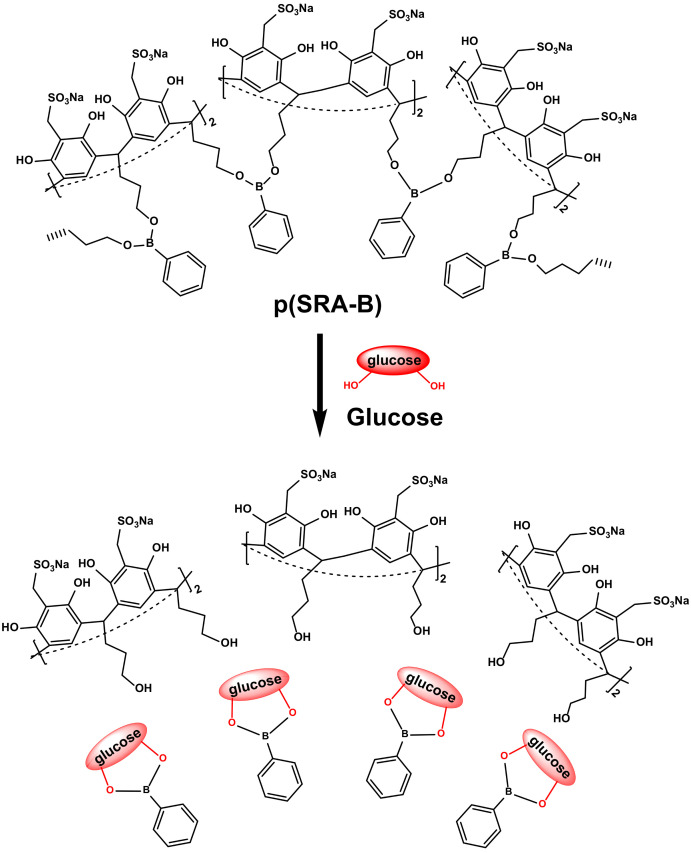
Glucose-binding induced dissociation of p(SRA-B) [166]. Reprinted with permission from *ChemPlusChem* **2019**, *84*, 1560–1566. Copyright 2019, Wiley.

In addition, sulfonated resorcinarene (SRA) molecules could interconnect to each other by phenylboronate bridges to obtain a glucose-sensitive nanocarrier (p(6SRA-5B)) for insulin delivery [166]. At normal glucose levels in blood plasma (5 mM), p(6SRA-5B) exhibited excellent stability, with insulin release not exceeding 10%. As glucose concentration increased (7.5–10 mM), the phenylboronate ester segments in p(6SRA-5B) competitively reacted with excess glucose, breaking the linkage between phenylboronic acid and SRA, thereby releasing the insulin from the cavity (Figure 16).

## 6. Pillar[n]arene-Based DDSs

**Figure 17:**
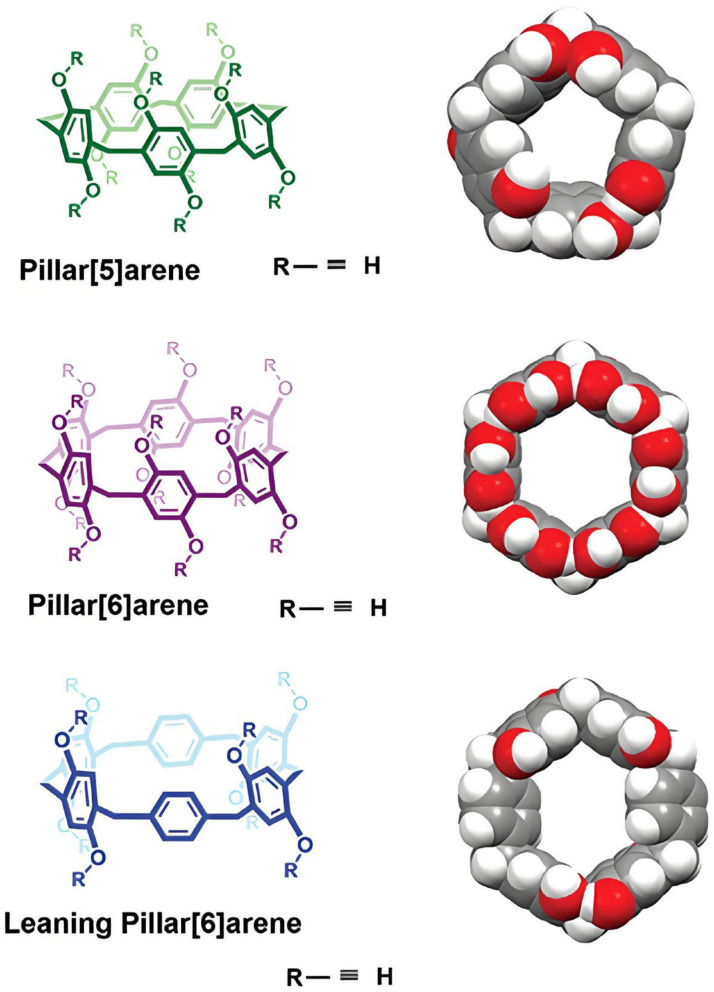
Molecular structures and space-filling models of P[5]A, P[6]A, and leaning P[6]A [168]. Reprinted with permission from *Adv. Mater.* **2020**, *32*, e2003263. Copyright 2020, Wiley.

Pillar[n]arenes (P[n]As) are a class of cyclic oligomers composed of hydroquinone or hydroquinone ether connected to the para-position of the benzene ring through a methylene bridge. It is a novel class of macrocyclic host molecules (Figure 17). P[n]A has hydrophobic and electron-rich cavities and rigid columnar structure, and is easy to be functionalized, making it a promising carrier for DDSs [167].

### 6.1. P[n]A-Based Inclusion Complexes

**Figure 18:**
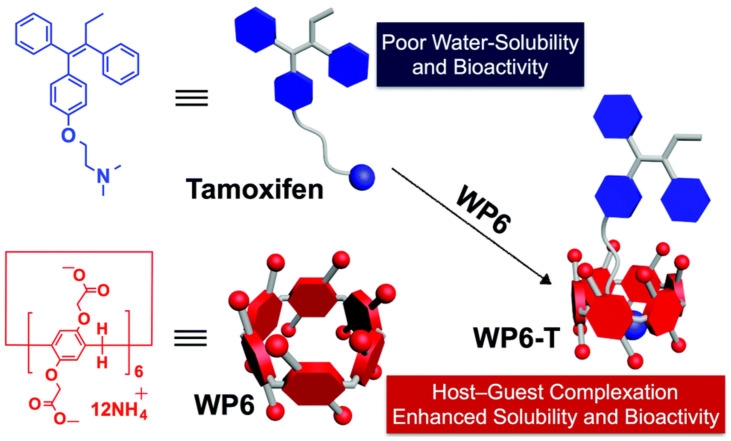
WP[6]A encapsulates tamoxifen to enhance its water solubility and biological activity [170]. Reprinted with permission from *Chem. Commun.* **2017**, *53*, 9749–9752. Copyright 2017, Royal Chemical Society.

P[n]As can increase the stability, solubility, and bioavailability of the encapsulated guest molecules. However, the water solubility of ordinary P[n]As is limited. The structure of both sides of P[n]As is easily modified, and most of the functionalized derivatives show good water solubility, low toxicity, and selective binding to the guests [169]. Among them, carboxyl-modified water-soluble P[n]As (WP[n]As) are widely used to bind various drug molecules. Huang and colleagues used WP[6]A to encapsulate anticancer drug tamoxifen to enhance the water solubility and bioactivity of tamoxifen (Figure 18) [170]. Zhang et al. reported that a WP[6]A–oxaliplatin inclusion complex exhibited higher bioavailability to cancer cells and lower cytotoxicity to normal cells than that of free drugs [171].

Similar to CB[n]s, P[n]As can also achieve detoxification function by encapsulating toxins [172]. WP[6]A has been shown to reverse the toxicity of paraquat in vitro [173]. It has also been proven to reduce various serious side effects of succinylcholine in mice, even reversing high-dose lethality [129].

Researchers have also coupled integrin α_v_β_3_-targeting peptide P1 with P[5]A (P1P5A) to successfully deliver P[5]A to tumor tissues [174]. More importantly, P[5]A exhibits high-affinity inclusion behavior with cationic polyamines (association constants of 10^5^–10^6^ M^−1^). Therefore, the trap P1P5A could induce apoptosis of tumor cells by influencing the polyamine biosynthesis pathway both in vitro and in vivo. Different from carrying anti-tumor drugs, the work reveals a quite new approach for suppressing tumor growth by using supramolecular macrocycles.

### 6.2. P[n]A-Based NDDSs

**Figure 19:**
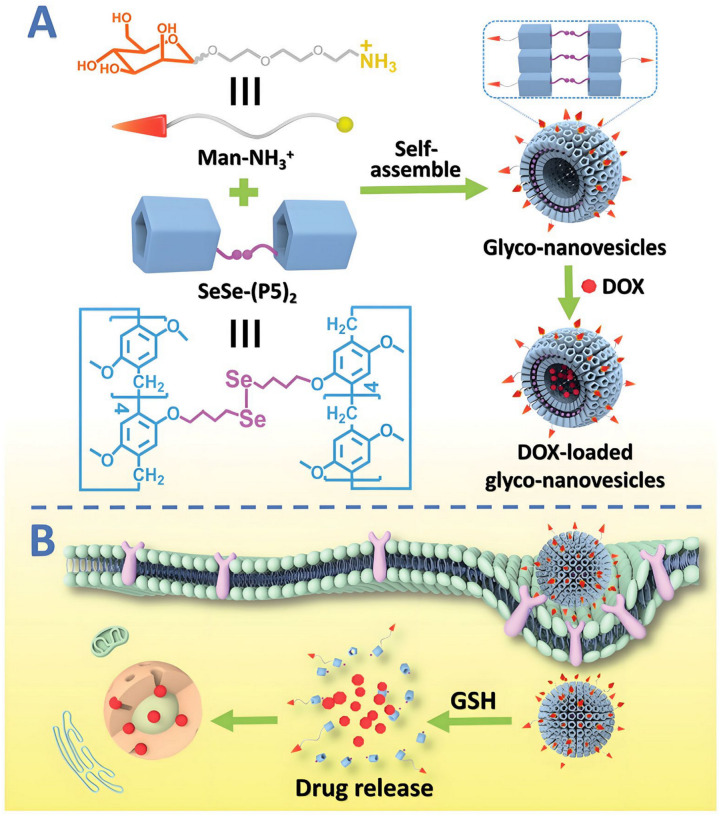
An illustration of the construction of supramolecular glycol-nanovesicles: SeSe-(P5)_2_⊂Man-NH_3_^+^ (**A**) and their GSH-responsive targeted chemotherapy (**B**) [176]. Reprinted with permission from *Chem. Commun.* **2020**, *56*, 10642–10645. Copyright 2020, Royal Chemical Society.

In addition to directly utilizing the encapsulation effect of P[n]As, many studies have also focused on loading drugs in supramolecular carriers based on P[n]As. For example, Xia et al. designed and synthesized a novel amphiphilic PEGylated P[5]A (EtP5-SS-PEG) containing disulfide and amide bonds. Through its host–guest interaction with perylenediimide, they self-assembled to construct a novel supramolecular nanocarrier with dual responsiveness to enzymes and glutathione [175]. Pei et al. also synthesized a diselenium-bridged P[5]A dimer [176]. By utilizing the host–guest recognition effect of the P[5]A cavity on cations, a mannose–NH_3_^+^ derivative is encapsulated into P[5]A to form amphiphilic molecules, which self-assemble to tumor microenvironment-responsive and cancer cell-targeted vesicles due to the diselenium bonds and mannose units, respectively. The DOX-loading vesicles further confirmed the effectiveness of the vector in enhancing chemotherapy effects (Figure 19).

**Figure 20:**
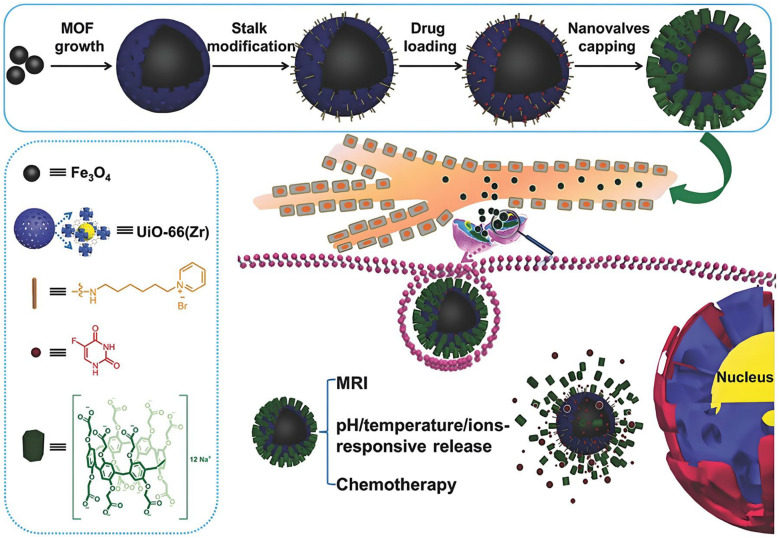
An illustration of the fabrication process and operation of WP[6]A-based theranostic MOF [179]. Reprinted with permission from *Small* **2018**, *14*, 1704440. Copyright 2018, Wiley.

Furthermore, P[n]A, as a “gatekeeper”, makes a porous drug loading system realize on-demand release. For example, P[5]A or its derivatives were coated on the surface of MSN modified with guests as supramolecular valves for drug delivery and controlled release [177,178]. Metal–organic frameworks (MOFs) are another kind of important porous material, which have broad application prospects in the field of biomedicine. Yang et al. used P[n]A-modified MOFs to construct a core–shell nanocomposite as a diagnosis and treatment system (Figure 20). The system uses Fe_3_O_4_ NPs as the core and UiO-66 MOF as the shell. The surface of UiO-66 MOF can be modified with a pyridinium cationic moiety, which has strong complexation with WP[6]A. Accordingly, the 5-fluorouracil-loaded core–shell nanocomposite can serve as a theranostic platform for multistimuli-responsive drug release and MRI-guided cancer therapy [179].

**Figure 21:**
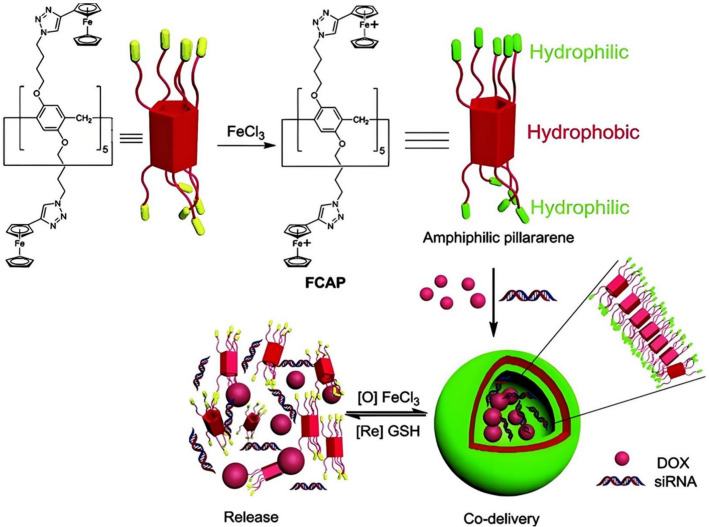
An illustration of the synthesis of the ferrocenium-capped amphiphilic P[5]A (FCAP), formation of cationic vesicles, and their GSH-responsive siRNA/DOX release [182]. Reprinted with permission from *Angew. Chem. Int. Ed. Engl.* **2014**, *53*, 13126–13130. Copyright 2018, Wiley.

In fact, P[n]As can also serve as gene transfection vectors. On the one hand, traditional gene carriers are limited in their application due to the toxicity of cationic lipids. Therefore, a pDNA lipid carrier based on a polycationic P[5]A derivative, anionic lipid DOPG, and zwitterionic lipid DOPE was designed and implemented [180]. When the concentration of polycationic P[5]A derivatives ≥15 μM, they are used as bridges between anionic lipids and pDNA; then, pDNA is effectively compacted by DOPG/DOPE mixed lipids. Finally, the transfection and cell viability experiments of COS-7 cells obtained a medium–high transfection level and good cell viability results. This suggests that P[n]As, especially cationic P[n]As, may open up a new and promising approach in the field of anionic non-viral gene vectors. On the other hand, macrocyclic amphiphilic Bola molecules have shown some potential for gene delivery, and highly symmetric P[n]A is an attractive candidate for preparing amphiphilic Bola molecules [181]. For example, Pei et al. reported a cationic vesicle based on ROS-responsive ferrocenium-capped amphiphilic P[5]A (FCAP). The vesicles can achieve co-delivery of DOX and siRNA, and the transfection efficiency is roughly the same as that of the classical siRNA transfection reagent Lipofectamine 2000 (Figure 21) [182].

**Figure 22:**
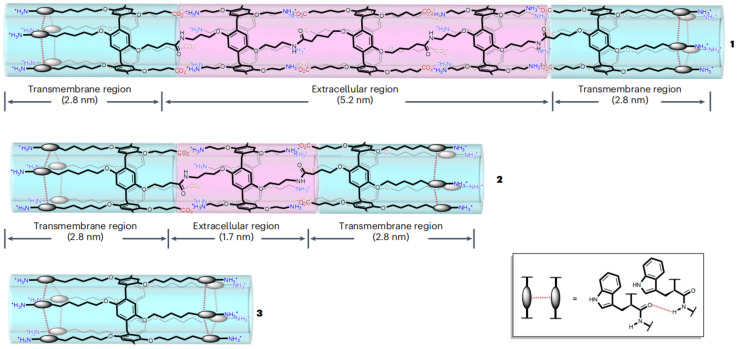
Chemical structures of artificial gap junctions (**1**) and (**2**) and the control channel (**3**) [183]. Reprinted with permission from *Nat. Chem.* **2024**, 1–9. https://doi.org/10.1038/s41557-024-01519-8. Copyright 2024, Springer Nature.

Another interesting study constructed an artificial intercellular gap junction channel from unimolecular tubular molecules consisting of alternately arranged positively and negatively charged P[5]A motifs [183]. The hydrophobic–hydrophilic–hydrophobic triblock structure allows the molecules to assemble and form stable tubular structures driven by electrostatic interactions (Figure 22). The length of the channels (10.8 nm) is long enough to stretch across the gap between two adjacent cells to form gap junctions, which could further mediate intercellular signals and reactive oxygen species transmission. This robust strategy for building artificial intercellular communication pathways is promising for further building artificial tissues or even artificial life.

## 7. Conclusions and Prospect

SDDSs based on macrocycles are becoming a bright new star in formulation strategies. Its significant advantage is that it can effectively enhance the solubility of insoluble drugs, then improve their bioavailability. Meanwhile, SDDSs significantly extend the retention time of the drug in the circulation, ensuring that the drug can continue to produce benefits. It is worth mentioning that based on the host–guest interaction, the formation of advanced spatial structures is skillfully induced. This provides new forms of drug delivery carriers, which is expected to bring revolutionary breakthroughs in the field of drug research and development. The responsiveness of the dynamic host–guest interaction ensures that the payloads can be accurately delivered to the targeted sites and effectively controls release. This could improve the efficacy of the drug and also reduce the potential risk to surrounding healthy tissues, providing a more accurate and safe approach for precise treatment. Therefore, SDDSs have shown great application potential in clinical medicine. Although nanomedicine such as Doxil^®^ and Onivyde^®^ have been approved for clinical use, their efficacy still needs to be improved. The potential immunotoxicity issues and high cost have become key factors limiting their widespread use.

We believe that there are still three urgent issues that need to be addressed in the SDDSs based on macrocycles:

I. The sensitivity and specificity of supramolecular systems towards lesion tissues or cells still need to be strengthened. At present, the system still has certain limitations in accurately identifying and locating specific lesion areas, which may result in unexpected drug distribution, thereby affecting treatment effectiveness. Therefore, future research needs to focus on improving the sensitivity and specificity of SDDSs to achieve more precise and effective drug delivery.
II. Due to the dynamic and weak nature of non-covalent interactions, the stability of host–guest complexes or assemblies is often challenged. This instability may lead to drug leakage during delivery, which may result in a series of side effects and expose patients to unnecessary health risks. Therefore, when designing and optimizing SDDSs, it is necessary to fully consider the shortcomings of non-covalent interactions, and take corresponding measures to improve the stability of the system to ensure that drugs can reach the target site safely and effectively. Designing macrocycles and guests that are capable of attaining biotin/(strept–) avidin level affinity in aqueous environments is an attractive and long-term subject.
III. There is another practical challenge; that is, the industrial quantitative production of macrocycles and supramolecular systems is difficult. It is crucial to achieve stable and efficient preparation processes for macrocycles and supramolecules in order to promote their widespread application in the pharmaceutical field. However, this process requires overcoming many technical difficulties and continuous exploration and innovation by researchers.

To sum up, in order to further promote the development of SDDSs based on macrocycles, we must strengthen the exploration of different macrocyclic molecular containers and in-depth studies of host–guest interactions. Based on this, research of SDDSs can be promoted to a new height, so that the stable supramolecular structure with high sensitivity would optimize the solubility of drugs, improve bioavailability, reduce the toxic side effects, and then successfully be applied to improve the precision of disease-targeted therapy and gene delivery. There is reason to believe that the field of SDDSs based on macrocycles will continue to bring us many remarkable innovations, and these new materials will lead to more advanced and efficient delivery systems and new therapies to make greater contributions to human health.
